# COMET IV meeting summary

**DOI:** 10.1186/1745-6215-16-S1-M1

**Published:** 2015-05-29

**Authors:** Elizabeth Gargon, Doug G Altman, Jane M Blazeby, Mike Clarke, Paula R Williamson

**Affiliations:** 1 University of Liverpool, Department of Biostatistics, Liverpool, UK; 2 University of Oxford, Centre for Statistics in Medicine, Oxford, UK; 3 University of Bristol, School of Social and Community Medicine, Bristol, UK; 4 Queens University Belfast, All Ireland Hub for Trials Methodology Research, Belfast, UK

## 

On two sunny days in Rome in November 2014, more than 200 people with an interest in core outcome sets (COS) from around the world gathered at The Pontificia Università Lateranense for the fourth meeting of the COMET Initiative. They came from 14 countries: Austria, Canada, Denmark, France, Germany, Ireland, Italy, Netherlands, Poland, Portugal, Romania, Switzerland, UK and USA. The programme was introduced by Liz Gargon (COMET Project Coordinator) and a summary of the coming days was provided by the chair of the COMET Management Group (Paula Williamson). Paula also presented the COMET strategic plan and provided a general update and progress report since the last meeting in Manchester in June 2013. Over the two days, the invited plenary talks were complemented by three workshops, 52 posters (50% more than the number at the 2013 meeting) and four contributed presentations.

On 19^th^ November, Roberto D’Amico (University of Modena) talked about the need for core outcome sets to improve evidence synthesis, and Silvio Garattini (IRCCS-Istituto di Ricerche farmacologiche Mario Negri) followed him by discussing the use of surrogate and composite endpoints. He underlined the differences between core sets and these end points and explained how the latter did not always examine the outcomes most important to patients. This became a prominent theme throughout the meeting and was the focus of a dedicated session on 20^th^ November focussing on patient engagement and involvement in COS development. A patient representative (Rosemary Humphreys – HOME Initiative) emphasised why core outcome sets are so important to patients, highlighting how patients looking for information on their conditions on the internet and elsewhere are confronted not only by the challenges of finding reliable information but, when they do, they might find themselves looking at trials that did not compare the same outcomes. She also stressed how doctors might know about the condition, while patients know about the impact. We also heard about the potential challenges of public involvement from the perspective of a COS developer (Iain Bruce, Royal Manchester Children's Hospital). Iain spoke about the practical, but essential, aspects of arranging face to face meetings and ensuring comprehensive involvement from all sections of a patient group or society, including ‘hard to reach’ groups. Heather Bagley, COMET Patient and Public Involvement (PPI) coordinator, concluded this session with a presentation on ‘Involving people and the COMET PPI strategy’, which outlines COMET’s public involvement objectives and initial plans for public involvement activities. This report is available at http://www.comet-initiative.org/resources/publicinvolvement.

In a session designed to turn the generalities of developing COS, into the specifics of individual projects, we also heard from three COS developers (Christian Apfelbacher, University of Regensburg; Finn Gottrup, Copenhagen Wound Healing Center, Bispebjerg University Hospital, and Alessandro Chiarotto, VU University) about the methods used and their experiences of developing COS. Discussion included the importance of defining the scope of the COS from the outset and thinking about implementation early on in the process. This provided the foundation for later sessions, including one on COS development methodology (Sara Brookes, University of Bristol, and Sanna Prinsen, VU University). We also learned more about the relevance of COS to systematic reviews, in particular Cochrane Reviews. Holger Schünemann (McMaster University) highlighted the links to the GRADE summary of findings tables, suggesting that COS can help reviewers decide on the important outcomes to present in their tables. Valerie Smith (Trinity College Dublin) presented a survey of outcomes in Cochrane Reviews, emphasising that consistency of reporting of outcomes across and between Cochrane Review groups is a challenge and that Cochrane needs to play its part by using COS in their reviews to help overcome this. The example of pain as an outcome that cuts across many of these Groups was explored further by David Tovey (Cochrane Library) and Peter Tugwell (University of Ottawa). It was clear that there is much to be done to help standardise the outcomes across and between Cochrane Groups, and COS might be a great starting point for this.

The final session focussed on the links between COS, COMET and other groups, such as the European Medicines Agency (Irmgard Eichler) and National Institutes of Health (Jerry Sheehan), and showed the potential role for COS in the development of regulatory guidelines. Sean Tunis (Center for Medical Technology Policy) reported on the first COMET network meeting in North America, held earlier in the year, where many organisations including FDA, AHRQ, NIH and PCORI came together to learn about the importance and relevance of COS, and to think more about the role that each of these organisations may play. Payers were highlighted as being stakeholders that had not been mentioned much through the meeting but were another group that might have an important role to play in the development and implementation of COS, due to the rising costs of healthcare and the pressures on them to spend money efficiently. Sean described how he will use COS and refer to COMET in guidance papers developed by the Green Park Collaborative. Perhaps the most importance message from this session was that we need to find ways to collaborate and add value to the work of these other organisations. This was exemplified by Khalid Khan (Barts and The London School of Medicine and Dentistry) who described the CoRe Outcomes in WomeN's health (CROWN) Initiative, a consortium of women’s health journals aiming to promote core outcome sets in all areas of the specialty, which now has around 70 different journals signed up and committed to the initiative.

COMET IV provided a unique opportunity for stakeholders from different environments to share their ideas and progress, and engage in discussion and debate. Participants shared their challenges, needs, solutions and resolutions. The meeting brought together key scientists and consumers responsible for developing and implementing COS, including trialists, systematic reviewers, health service users, clinical teams, journal editors, trial funders, policy makers, trials registries and regulators. There was resounding support for the COMET Initiative and unanimous agreement that COS have a vital role to play in the future of clinical trials and health research. Since COMET IV, we have already had follow up contact with people and groups from the meeting. One example is the European Commission Initiative in Breast Cancer (ECIBC), and we hope that our days in Rome will be the catalyst for much successful and productive collaboration in the future. It was clear from the enthusiasm and dedication shown throughout COMET IV that there is a continued commitment to COS development and application.

The slides from COMET IV presentations can be viewed at: http://www.comet-initiative.org/events/FourthCometMeeting

